# A neuropathology career nudged along by circumstances

**DOI:** 10.17879/freeneuropathology-2026-10215

**Published:** 2026-10-15

**Authors:** Lawrence A. Hansen

**Affiliations:** Department of Pathology, University of California San Diego, La Jolla, CA, USA

**Keywords:** Neuropathology, Dementia, Alzheimer disease, Reflections, UCSD, Autobiography

*I claim not to have controlled events, but confess plainly that events have controlled me*

Abraham Lincoln

## Introduction

There is nothing wrong with looking back, said Lot’s wife. When some end-stage neuropathologists reminisce, they can reflect upon careers driven by ambition. But others feel compelled to acknowledge the role played by seemingly random circumstances, perhaps better termed dumb luck, in their academic success. This essay of the latter sort is intended to inspire directionless pathology residents with no aptitude for research and a work ethic skewing towards the slothful who wonder if somehow they too could live the good life of an academic neuropathologist.

## Part one: Stumbling out of the starting gate and the life-changing lunch

My sole motivation for going to medical school was to become a psychiatrist. But during my psychiatry internship, I quickly learned that the psychotherapeutic approach to mental illness which had inspired that career choice was considered in contemporary psychiatry as largely akin to balancing the four humors, and that what patients really needed was a well-stocked drug store.

Having decided not to continue in psychiatry, unemployment was fast approaching towards the end of that first postgraduate year (PGY 1) when I happened to have lunch with a classmate from medical school who had gone into pathology, about which my ignorance was total. Conversing with her conjured up vague memories of an enjoyable medical school pathology course, but I arrived at my professional Damascus Road when she told me that pathology residency required no in-house overnight call. Not only that, but since I could pursue combined anatomic and clinical pathology (AP/CP) training at the same institution where I had fumbled through medical school and my psychiatry internship, Loyola University Chicago’s Stritch School of Medicine, I would not even have to get a new parking permit. The only downside was that I had no idea what a pathologist did for a living and no desire to be one, but four years of employment looked good at the time.

## Part two: Huh. So this is pathology

The anatomic side of AP/CP training proved unexpectedly interesting, unbeknownst to me sowing the seeds for a neuropathology career to follow. Clinical pathology years streaking agar plates in microbiology, searching for the anion gap in clinical chemistry, and puzzling over antibody panels in the blood bank also passed painlessly enough. My obtuseness during residency about medical academe was embarrassingly revealed during a conversation with my department chair. He expressed regret that I was not academically inclined, which baffled me, so I responded that uninterrupted schooling since age five ought to be academic enough to impress anyone. He disguised his dismay at my ignorance and had to enlighten me about what the letters CV stood for. I ignored his advice about writing papers. The four AP/CP years passed quickly and, with me still passive about a pathology career, unemployment loomed again. Circumstances/dumb luck came to my aid once more when an ad for an open neuropathology (NP) fellowship position was thumbtacked to the residency room bulletin board. This was intriguing since the neuropathologist at my institution was a cool, charismatic guy who drove a Mercedes-Benz convertible, threw parties for the residents at his opulent home with an indoor swimming pool, and never left work later than 3:30 pm. Thus inspired, I applied for the fellowship, only learning years later that my neuropathologist’s enviable lifestyle might have had something to do with a wealthy ex-wife.

## Part three: Southern moonlight and magnolias

In response to my application, the Emory University NP fellowship director called and invited me to Atlanta to interview for the position. Because I would soon be living on my meager savings from residency if I didn’t get the job, I asked the director if he could decide yes or no based on my application alone, and save me the expense of a trip that I couldn’t afford and that might end in rejection anyway. He responded memorably by telling me that I looked OK on paper but if I turned out in person to be a jerk (that wasn’t the exact word he used, but you get the idea), then that would not be his fault, would it? Intrigued by his candor, I made the trip and went through the interviews. Being the only applicant probably helped me survive the rigorous just-don’t-be-a-jerk screening, and the position was offered.

Thus began three very pleasant years of NP fellowship training. The extra year was graciously granted by Yoshio Takei, a brilliant neuropathologist, tireless advocate for his fellows’ welfare, and dynamic, if sometimes overpowering, personality, when he realized I hadn’t had the foresight to line up a job as the second year was ending. Another Emory neuropathologist, Suzanne Mirra, an expert diagnostician, electron microscopist extraordinaire, and supportive, nurturing friend, was then in the process of creating the Consortium to Establish a Registry for Alzheimer Disease (CERAD), the academic anlage of today’s National Alzheimer's Disease Coordinating Committee. Unbeknownst to me at the time, while she was imparting to me her expertise in neurodegenerative neuropathology, Sue was buying me a ticket to ride the Alzheimer disease (AD) funding gravy train in later years.

Even the largess of the Emory NP department had its limits, so as the third year of my two-year fellowship was nearing its end, the only secure employment I had lined up for July 1985 was working the parking lot kiosk at nearby Georgia Baptist Hospital. The circumstances/dumb luck pattern reemerged when Dr. Gary Pearl, another stellar Emory neuropathologist closer to my age and both a mentor and academic big brother figure, brought me an ad he saw in *Science* magazine for a Fellowship in Geriatric Neuropathology and Dementia at the University of California San Diego (UCSD). Reasoning rightly that I was a “the journey is the destination” kind of guy, largely coasting through life, Gary figured that another fellowship (PGYs 9–10) would keep me off the streets a while longer and, being in California, I could learn to surf. Being again the sole applicant, I turned out to be the most qualified candidate and got the job.

## Part four: Go west young man, go west

The UCSD fellowship was in the Department of Neurosciences, not the Department of Pathology. It was designed to provide fertile academic soil in which a creative young neuropathologist’s research career could germinate. Prior to my arrival at UCSD, the internationally renowned neuropathologist Dr. Robert Terry had for decades been fostering the careers of young research-oriented neuropathology fellows. After securing enough grant funding to provide them with abundant laboratory resources, he then employed what he termed “judicious neglect” in getting out of the fellows’ way as their scientific creativity blossomed.

That mentoring strategy fit me like a glove fits an elbow. Having no original ideas to pursue but feeling the pressure to publish, I resorted to the scientific bottom-feeding of lesion quantification and started enumerating plaques, tangles, and Lewy bodies in dementia brains and controls coming from the UCSD Alzheimer's Disease Research Center (ADRC). Frozen hemispheres from the brains thus quantified were then handed off to ADRC researchers in the gels and blots business, and the deceptively objective-appearing lesion counts sent to ADRC neurologists and neuropsychologists who got busy with clinico-pathologic correlations. Their research publications all included me as a co-author and my lengthening CV began to resemble that of a real neuroscientist, all because I could count between zero and fifty. But after two years, counting started to get old and I developed a nostalgic affection for the stimulating variety of surgical pathology as I remembered it from five years prior. So, for PGY 11 I detoured away from neuropathology into a surgical pathology fellowship to brush up my diagnostic skills preparatory to finally getting a real job as a worker-bee surgical pathologist somewhere.

## Part five: Motor City madness

That delusion shattered on the first day of my surgical pathology fellowship at William Beaumont Hospital in Royal Oak, Michigan, just outside Detroit. With rising panic, I searched the stack of thirty biopsies set before me seeking in vain a meningioma, schwannoma, pituitary adenoma, glioblastoma, or brain metastasis, something, anything I could confidently diagnose. How could there even BE thirty consecutively accessioned cases with no neuropathology? It was then I remembered why, five years prior at the end of the AP/CP residency, I had decided on fellowshipping rather than confronting the challenges of a practicing pathologist. Regret is illumination come too late; I had signed the contract and so I put in the year, which only served to reinforce that sickening first-day insight about my unsuitability for surgical pathology as a career.

As that year neared its end, unemployment approached once more, like Inspector Javert closing in on Jean Valjean in Victor Hugo’s *Les Misérables*. I wondered if the parking lot kiosk job at Georgia Baptist might still be open, but then decided to look into a PGY 12 fellowship year in forensic pathology or even, God help me, cytology. I was spared such desperate measures when, deus ex machina, Dr. Terry called to offer me a faculty job.

## Part six: Back to the future

It turned out that no one could follow my act as a Geriatric Neuropathology and Dementia Fellow. That is to say, nobody applied after I left for Michigan, and the UCSD Department of Neurosciences still needed someone to cut their ADRC brains and count plaques, tangles, and Lewy bodies. Needing that, they offered to appoint me as an assistant professor on a six year up-or-out research track to keep cutting and counting. I had no illusions that my kind of research would warrant promotion to associate professor and tenure at the end of the six years, but that would still be longer than I had ever previously held a job, and would get me to middle age before heading for the kiosk. The downside was that even my brain needed more stimulation than counting to fifty, and I lacked the scientific creativity to provide it for myself. Dr. Terry very accommodatingly cut a deal with the Department of Pathology, where he also held an appointment, to let me participate in the clinical neuropathology service, thus allowing me to recapture a physician identity to complement that of a lame researcher.

The clinical NP service at UCSD was then largely under the auspices of Clayton Wiley, MD, PhD. He was the departmental wunderkind who had finished the NP fellowship there a few years earlier and embarked on a dazzlingly successful career as a true physician-scientist. Besides signing out the NP biopsies and autopsy brains, and teaching residents, medical students, and an NP fellow, he was running a research enterprise at the time after having secured grant funding and established his own lab. Being that busy, he was receptive to the idea of sharing the clinical service. Henry Powell, a more senior neuropathologist who was head of the NP division in the Department of Pathology and ran the Veterans Administration (VA) NP service, also welcomed me warmly and was invaluable in advancing my career at UCSD.

So by July 1988, my 11 years of pinballing between residency and fellowship bumpers in Chicago, Atlanta, San Diego, and Detroit had somehow landed me in a neuropathology career catbird’s seat. The clinical duties and interactions with fellow pathologists, neurosurgeons, and neurologists satisfied my need to feel like a real doctor. Teaching medical students, residents, and fellows was both a joy and a sanctioned outlet for pathological narcissism. Ongoing cutting and counting of ADRC brains were generating such a steady stream of co-author publications that I began to fantasize about clearing the hurdle of promotion to associate professor and having a career longer than six years.

The fly in the ointment was that UCSD was a self-proclaimed prestigious (revealing etymology of that word) research-intensive university, and its guardians of tenure would be dismissive of co-author publications however numerous. They would instead insist upon impactful first-author publications, impact being measured largely by citation index, in choosing the up or the out option when deciding if an assistant professor should be promoted to associate or culled from the herd. That posed a problem for someone demonstrably lacking scientific creativity. Serendipitously, though, I had written several papers which were oft-cited, but that too was the result of circumstances/dumb luck.

Back in the Emory days, when Sue Mirra was getting CERAD up and running, she showed me a dementia brain with a neuropathologic trifecta: AD plaques and tangles, Parkinson disease Lewy bodies, and Creutzfeld-Jakob disease-like spongiform vacuolization confined to the entorhinal cortex. In cutting and counting hundreds of UCSD ADRC brains, I came across a substantial cohort of similar cases, and when we retrospectively reviewed neurologic exams and psychometric data, we found that these patients were clinically distinguishable from those with pure AD pathology. No claim to brilliance or precedence about that. The same association of Lewy bodies with dementia rather than just motor parkinsonism was being reported from many of the other ADRCs that had sprouted like mushrooms following financial rain pouring from the National Institutes of Health (NIH) tasked with promoting AD research. My publications’ lofty citation indices merely reflected the fact that each center publishing the same finding employed its own idiosyncratic nomenclature and felt compelled to take a shot at competing labels as undesirable designations. The back-and-forth nosologic debates were as lacking in scientific substance as a Swiftian war between Lilliput and Blefuscu over which end of an egg to open, but they generated lots of citations, getting me over the six-year hump with a promotion and tenure. This final infusion of Forrest Gump luck extended my academic neuropathology lifespan from six to thirty-eight years.

## Coda

The retrospective implausibility that a disillusioned soon-to-be-unemployed psychiatry intern with no interest in or capacity for research and a blissful ignorance about all academic things would end up enjoying a 38-year career in neuropathology inspires musings about the imponderable. Was Seneca right that fate guides the willing and drags the unwilling, or Hamlet when he opined that there is a Divinity that shapes our ends, rough-hew them how we will? Maybe, or maybe dumb luck is just dumb luck. Either way, I gratefully acknowledge my debts to Yoshio Takei, Sue Mirra, Gary Pearl, Bob Terry, Clayton Wiley, and Harry Powell, each of whom materialized with impeccable timing, like Athena guiding Odysseus, to steer me further along a path that, but for their interventions, would have dead-ended.

**Figure 1:**
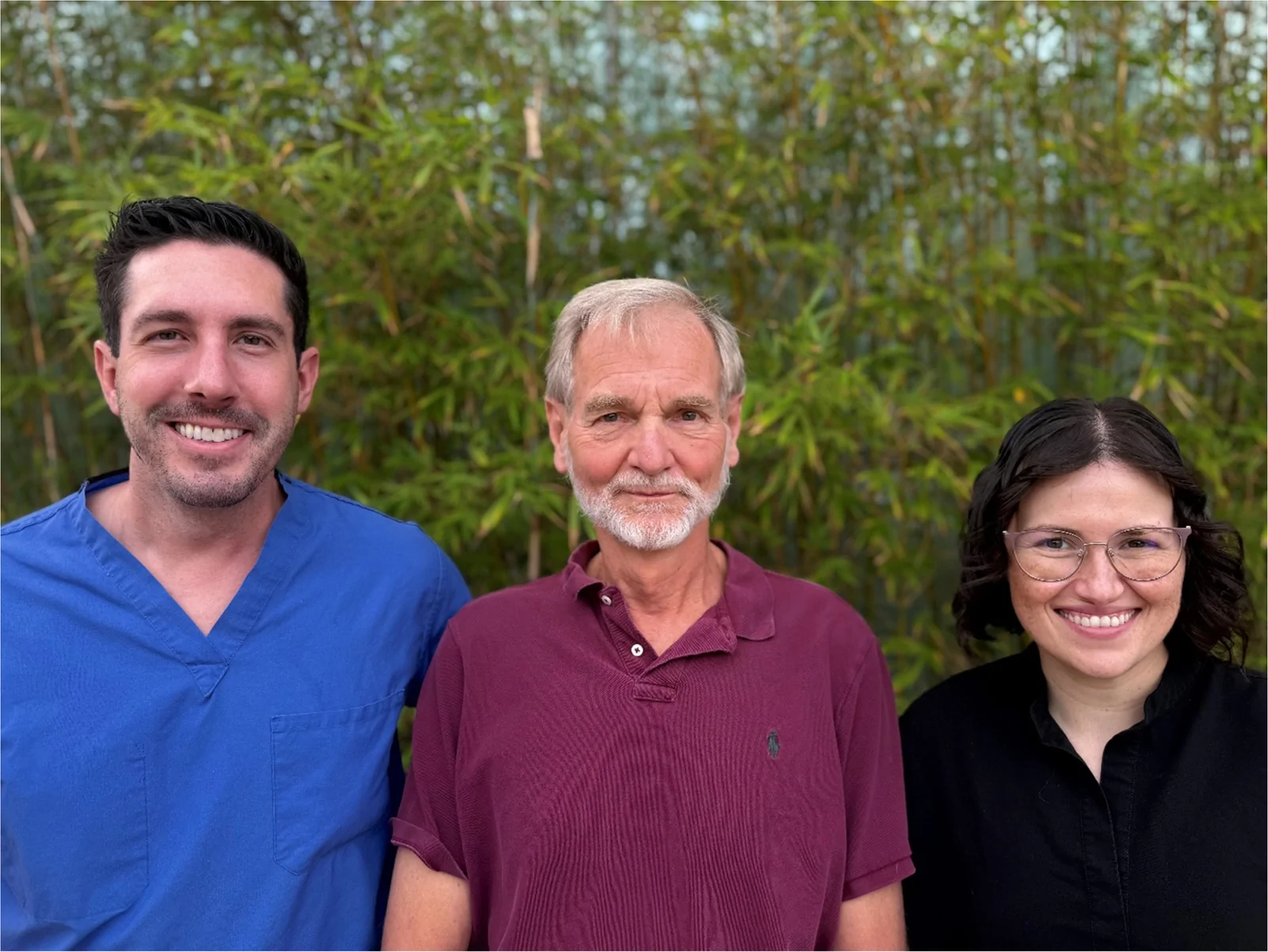
The author (old man in the middle) on the cusp of retirement with two of his long-suffering former fellows and current faculty colleagues, Connor Zuraski and Vanessa Goodwill, who graciously enabled his professional Long Goodbye.

